# Numerical simulation of the nanofluid flow consists of gyrotactic microorganism and subject to activation energy across an inclined stretching cylinder

**DOI:** 10.1038/s41598-023-34886-2

**Published:** 2023-05-12

**Authors:** Hakeem A. Othman, Bilal Ali, Sidra Jubair, Musawa Yahya Almusawa, Sayed M. Aldin

**Affiliations:** 1 Department of Mathematics, AL-Qunfudhah University College, Umm Al-Qura University, Mecca, Kingdom of Saudi Arabia; 2 School of Mathematics and Statistics, Central South University Changsha, Changsha, 410083 China; 3 School of Mathematical Science, Dalian University of Technology, Dalian, 116024 China; 4 Department of Mathematics, Faculty of Science, Jazan University, 45142 Jazan, Saudi Arabia; 5 Faculty of Engineering, Center of Research, Future University in Egypt, New Cairo, 11835 Egypt

**Keywords:** Computational science, Mathematics and computing

## Abstract

The current study examines the numerical simulation of the nanoliquid boundary layer flow comprising gyrotactic microbes with mass and energy transmission across a stretching inclined cylinder. The consequences of chemical reaction, heat generation/absorption, buoyancy force and Arrhenius activation energy is also considered on the nanofluid flow. The flow mechanism has been modeled in the form of system of nonlinear partial differential equations (PDEs). That system of PDEs is further transform into the dimensionless set of ordinary differential equations (ODEs) through the similarity substitutions. The obtained set of differential equations are numerically computed through the parametric continuation method (PCM). The effects of the distinct physical constraints on the energy, velocity, mass and the motile microbe profiles are discoursed and evaluated through Tables and Figures. It has been noticed that the velocity curve drops with the influence of inclination angle and Richardson number, while enhances against the variation of curvature factor. Furthermore, the energy field boosts with the upshot of inclination angle and heat source term, while declines with the influence of Prandtl number and Richardson number.

## Introduction

There are various technical and manufacturing fields where the fluid flow through a stretchable surface has significant implications. such as the abstraction of elastic sheets, compression activities, the production of paper and fiber swirls, and so forth. Crane was the first to investigate the fluid flow across an extending flat sheet^1^. Saeed et al.^2^, Bilal et al.^3^ and Giul et al.^4^ have reported an ideal case study for assessing unstable nanoliquid across a strained sheet. Bilal et al.^5^ computationally presented the idea of heat flow and radiation impact on a stretchable sheet of hybrid nanofluid with tiny particles. Wang and Ng et al.^6^ analyzed the flow pattern with velocity slip condition across the inclined stretchable cylinder. They found from this research that slide effects have a strong influence on skin friction and velocity distribution. Stretching cylinder has applications in refrigeration systems, crystal growth, and glass fibre manufacturing^7^. Ramesh et al.^8^ documented the effect of a dusty Casson fluid flowing across an extending cylinder over the convective state and heat radiation. Nanoliquid flow across an inclined extending surface cylinder has attracted the curiosity of researchers, as evidenced by^9–15^.

The scientific community's focus has recently moved to the study of gyrotactic bacteria in boundary layer flows. A microbe is a biological thing with the capability to evolve, reproduce, respond to environmental stimuli, and maintain in an organized order. Any breathing organism like animals, fungus, or bacterium will be measured entities in this approach. These species can be classified in many ways. Some other use is microbial enhanced oil recovery, during which micronutrients and microorganisms are added to gasoline layers to reduce permeability differences. Aziz et al.^16^ predicted about the bioconvective flow of motile microorganisms and nanoparticles inside a permeable medium. They discovered that bio-convection factors had a major impact on the transmission rate of motile bacteria in the flow. Moreover, gyrotactic microorganisms can increase the flow stability of nanofluids^17^. Makinde and Animasaun^18^ inspected the impact of a magnetic flux, irregular thermal radiation, and a relatively homogenous biquadratic autocatalysis chemical change on an electrolyte solution ${\text{Al}}_{2} {\text{O}}_{3}$ and Ferrofluid that included the gyrotactic-microorganism on the upper vertical surface of a parabolic reflector. The cumulative upshot of Riemann slip and magnetization effect on temperature and mass flow of motile microorganisms in a water-based microchannel across a upright sheet was calculated by Khan et al.^17^. Furthermore, Khan et al.^19^ revealed related research for which they explored the free convective flow in the highly permeable medium by taking Copper nanoparticle across a stretched surface. A mathematical model established by Zuhra et al.^20^ to explore the flow and temperature distribution along a straight solid surface under the impact of autocatalysis nuclear reaction and motile microorganisms. Bioconvection influenced by the hydro-magnetic flow comprising of nanostructured materials and motile microbes past a porous material was deliberated by Mutuku et al.^21^. This analysis was enhanced by another report, Mahdy^22^, which examined the flow through mixed convection with the influence of thermal boundaries layers. Khan et al.^23^ scrutinized the fluid flow with the impacts of gyrotactic motile microbes, reaction temperature with the help viscous dissipation. As a result of such application domains, Beg et al.^24^ investigated bioconvection in thin film microchannel that included the variable viscosity, gyrotactic microorganisms and nanostructured materials using a particular nanoliquid model, which itself is critical for sophisticated bioconvection patterns appropriate to real life. However apart from these, the scientific community has used the bio-convective rheological behavior of nanomaterial associated to gyrotactic microbes in a wide range of projects^25–31^ and corroboration therein.

With the help of dissolving nanoparticles with a size range of (10–100 nm) in conventional heat transfer fluids, a new and sophisticated form of heat transfer has been generated. Nanofluids have significant applications in transportation and bio-medical (cancer treatments, nano-drug delivery) projects^32^. Maxwell was the first to show how increasing the volume proportion of rock-solid particles might show the likelihood of thermal conductivity developing in solid materials^33,34^. This leads to the term "nanofluid" being used to define a new type of fluid that has increased thermal conductivity and suspension consistency. Choi^35^ conducted the first investigation on the term "nanofluid" and found that it refers to liquids that are embedded in nanomaterials. Both at the macro and micro levels of heat transfer, conventional fluids like water, plasma, fuel oils, etc. play a vital role. However, these fluids have low heat transfer characteristics, which is one of their disadvantages. The idea of dissolving nano atoms in the base fluid is employed by academics and scientists to augments the energy transmission properties of the normal fluids^36–38^. Solid particles have superior heat transfer capabilities compared to ordinary fluid. Nanoparticles are in medicine delivery, energy conservation, and freezing capability. For this reason, the majority of movement in our world is caused by mass transfer. There are numerous of applications of mass transfer. It demonstrates how important it is to many engineering, industrial, semiconductor, solar, bio separations, metallurgical, and medal-winning areas. The principal applications of mass transfer occurrences in biomedicine include pharmacokinetics analysis, drug metabolism in the body, tissues manufacturing, containing the creation of artificial organs and catalytic converters in vehicles. Studies have been conducted taking into account the characteristics of the suspension of nano-sized solid particles. For example, Eastman^39^ had shown a 40 percent enhancement in the thermal conductivity. Raja et al.^40^ described the nanoliquid flow consist of MWCNTs. Raja et al*.*^41^, and Mohyud-Din et al*.*^42^ discovered that increasing heat conductivity and viscosity are caused by nanomaterial. They also observed that nanofluids are stronger and do not create blockage. The impact of nanoparticles in CuO, ethylene, and water was studied by Khedkar et al.^43^. When ${\text{Fe}}_{{3}} {\text{O}}_{{4}}$ nanoparticles were recycled due to which Kerosene oil's heat transfer rate increased by 30%, according to Parekh and Lee^44^. When a copper plate is put on a metal sheet, there will be some diffusion of molecules from both surfaces. Raja et al.^45^ has studied the nanofluid flow comprised of Au and MWCNTs. Using the Jeffrey model, Aiza et al.^46^ examined the temperature production in mixed convective Poiseulle movement caused by molybdenum disulfide. They used the perturbation approach to get approximations of the results for the energy and velocity curves. They also emphasized that the velocity of nanoliquid reduces as the volume percentage of particles rises. Zin et al.^47^ mentioned the effect of Ag nano particulates on Jeffrey fluid. After nanoparticles are evenly dispersed throughout the fluid, they showed how the rate of heat transmission may vary greatly. Some related studies may be found in Ref.^48–59^.

The purpose of the present analysis is to estimate numerically the nanoliquid boundary layer flow comprising gyrotactic microbes with mass and energy transmission across a stretching inclined cylinder. Additionally, the consequences of chemical reaction, heat source, buoyancy force and Arrhenius activation energy is also considered on the nanofluid flow. The flow mechanism has been modeled in the form of system of nonlinear PDEs. That system of PDEs is further transform into the dimensionless set of ODEs through the similarity substitutions. The obtained set of differential equations is numerically computed through the PCM. Furthermore, the phenomenon has been formulated, analyzed, solved and discussed in the coming sections.

## Mathematical formulation

**Figure 1:**
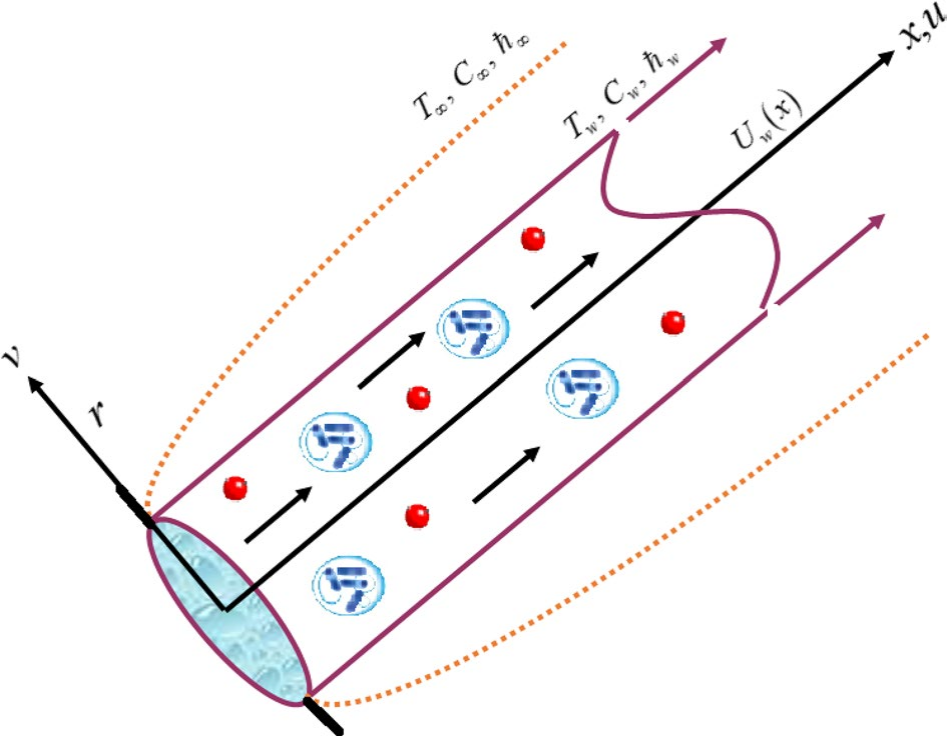
Physical sketch of the fluid flow.

We assumed a laminar, mixed convection and fixed density flow independent of time. The nanoliquid comprises of gyrotactic microbes across an elongating inclined cylinder. Horizontally the cylinder is extended with velocity $U_{w}$ and has radius *a* as presented in Fig. 1. Here, $T$ $T_{w}$ and $T_{\infty }$ is the fluid, surface and ambient temperature. The cylinder surface is exposed to $Q_{0}$ (heat source). The gravity force and difference of temperature between the surrounding and cylindrical surface creates the buoyancy force. Neglecting the external forces and pressure gradient, the basic equations are stated as^60,61^:

1 $$\frac{\partial }{\partial x}\left( {ru} \right) + \frac{\partial }{\partial r}\left( {rv} \right) = 0,$$

2 $$\begin{gathered} \rho_{f\infty } \left( {u\frac{\partial u}{{\partial x}} + v\frac{\partial u}{{\partial r}}} \right) - \mu \left( {\frac{1}{r}\frac{\partial }{\partial r}\left( {r\frac{\partial u}{{\partial r}}} \right)} \right) - \left( {1 - C_{\infty } } \right)\rho_{f\infty } \beta g\left( {T - T_{\infty } } \right)\cos \alpha_{1} + \left( {\rho_{p} - \rho_{f\infty } } \right) \hfill \\ g\left( {C - C_{\infty } } \right)\cos \alpha_{1} + \left( {\rho_{n\infty } - \rho_{f\infty } } \right)g\gamma_{1} \left( {n - n_{\infty } } \right)\cos \alpha_{1} = 0, \hfill \\ \end{gathered}$$

3 $$\begin{gathered} u\frac{\partial T}{{\partial x}} + v\frac{\partial T}{{\partial r}} = \frac{v}{\mu }\frac{1}{r}\left( {\frac{\partial }{\partial r}} \right)\left( {r\frac{\partial T}{{\partial r}}} \right) + \tau \left[ {D_{B} \left( {\frac{\partial T}{{\partial r}}\frac{\partial C}{{\partial r}} + \frac{\partial T}{{\partial x}}\frac{\partial C}{{\partial x}}} \right) + \frac{{D_{T} }}{{T_{\infty } }}\left( {\left( {\frac{\partial T}{{\partial r}}} \right)^{2} + \left( {\frac{\partial T}{{\partial x}}} \right)^{2} } \right)} \right] \hfill \\ + Q_{0} \left( {T - T_{\infty } } \right), \hfill \\ \end{gathered}$$

4 $$u\frac{\partial C}{{\partial x}} + v\frac{\partial C}{{\partial r}} = \frac{{D_{B} }}{r}\left( {\frac{\partial }{\partial r}} \right)\left( {r\frac{\partial C}{{\partial r}}} \right) + \frac{{D_{T} }}{{T_{\infty } }}\frac{1}{r}\frac{\partial }{\partial x}\left( {r\frac{\partial C}{{\partial r}}} \right) - k_{r}^{2} \left( {C - C_{0} } \right)\left( {\frac{T}{{T_{\infty } }}} \right)^{n} \exp \left( { - \frac{{E_{a} }}{\kappa T}} \right),$$

5 $$u\frac{\partial n}{{\partial x}} + v\frac{\partial n}{{\partial r}} = \frac{{D_{n} }}{r}\frac{\partial }{\partial r}\left( {r\frac{\partial n}{{\partial r}}} \right) - \frac{{b_{c} W_{c} }}{{\left( {C_{w} - C_{\infty } } \right)}}\frac{1}{r}\frac{\partial }{\partial r}n\left( {r\frac{\partial C}{{\partial r}}} \right)\,.$$

The boundary conditions are^60,61^:

6 $$\left. \begin{gathered} u = U_{0} \left( {x/l} \right) = U_{w} ,\,\,\,T = T_{w} ,\,\,n = n_{w\,\,} ,\,\,\,v = 0,\,\,\,C = C_{w} \,\,\,at\,\,\,\,r = a, \hfill \\ u \to 0,\,\,\,C \to C_{\infty } ,\,\,\,v \to 0,\,\,\,T \to T_{\infty } ,\,\,\,\,n \to n_{\infty } ,\,\,\,as\,\,\,r \to \infty . \hfill \\ \end{gathered} \right\}\,$$

here $u$ and $v$ components of the velocity. $\rho_{f} ,\,\,\rho_{n} ,\,\,\rho_{p}$ is the fluid, microorganisms and nanoparticles density. $k_{r}^{2}$ is the chemical reaction of second order, C is the concentration of the nanoparticles, $E_{a}$ is the activation energy. Furthermore, $\beta$,$g$, $\gamma_{1}$, $\alpha_{1}$ and $n$ is volume expansion, gravity acceleration, angle of inclination, microorganisms average volume and motile microbe density respectively, $\tau$, $D_{B}$, $D_{T\,}$, $D_{n}$, $b_{c}$ and $W_{c}$ is heat capacitance, Brownian diffusion, thermophoresis factor, microorganism diffusivity, Chemotaxis term and maximum cell floating velocity. The similarity variables are:

7 $$\eta = \frac{{r^{2} - a^{2} }}{2a}\,\sqrt {\frac{{U_{W} }}{vx}} \,,\,\,\psi = a\sqrt {vU_{w} x} f\left( \eta \right),\,\,\hbar = \frac{{n - n_{\infty } }}{{n_{w} - n_{\infty } }},\,\,\,\phi \left( \eta \right) = \frac{{C - C_{\infty } }}{{C_{w} - C_{\infty } }},\,\,\theta \left( \eta \right) = \frac{{T - T_{\infty } }}{{T_{w} - T_{\infty } }}.$$

Incorporating Eq. (7) in Eqs. (1) to (5):

8 $$\left( {2\gamma \eta + 1} \right)f^{\prime\prime\prime} + \left( {2\gamma + f} \right)f^{\prime\prime} - f^{{\prime}{2}} + Ri\left[ {\theta - N_{r} \phi - R_{b} \hbar } \right]\cos \alpha_{1} = 0$$

9 $$\left( {2\gamma \eta + 1} \right)\theta^{\prime\prime} + \left( {2\gamma + Prf} \right)\theta^{\prime} + \left( {2\gamma \eta + 1} \right)Pr\left[ {N_{B} \phi^{\prime} + N_{T} \phi^{\prime}} \right]\phi^{\prime} + Pr\zeta \theta = 0$$

10 $$\left( {2\gamma \eta + 1} \right)N_{B} \phi^{\prime\prime} + N_{B} \left( {2\gamma + Scf} \right)\phi^{\prime} + N_{T} \left( {\left( {2\gamma \eta + 1} \right)\theta^{\prime\prime} + 2\gamma \phi^{\prime}} \right) - Kr\left( {1 + \varepsilon \delta } \right)^{n} \phi \,\,\exp \left( { - \frac{E}{1 + \varepsilon \delta }} \right) = 0$$

11 $$\left( {2\gamma \eta + 1} \right)\hbar^{\prime\prime\prime} + 2\gamma \hbar^{\prime} + Lb\,Pr\hbar^{\prime} - Pe\left[ \begin{gathered} \sigma \gamma \phi^{\prime} + \sigma \left( {2\gamma \eta + 1} \right)\phi^{\prime\prime} + \gamma \phi^{\prime}\hbar + \hfill \\ \left( {2\gamma \eta + 1} \right)\phi^{\prime\prime}\hbar \, + \left( {2\gamma \eta + 1} \right)\phi^{\prime}\hbar^{\prime} \hfill \\ \end{gathered} \right] = 0$$

With boundary Conditions

12 $$\begin{gathered} f\left( 0 \right) = 0,\,\,\,f^{\prime}\left( 0 \right) = 1,\,\,\,\hbar \left( 0 \right) = 1,\,\,\,\theta \left( 0 \right) = 1,\,\,\,\phi \left( 0 \right) = 1\,\,\,\eta \to \infty : \hfill \\ f^{\prime} \to 0,\,\,\,\theta \to 0,\,\,\,\phi \to 0,\,\,\,\hbar \to 0 \hfill \\ \end{gathered}$$

where $\gamma = \frac{1}{a}\sqrt {\frac{vl}{{U_{0} }}}$ is the curvature parameter,$Ri = Gr_{x} /{\text{Re}}_{x}^{2} = g\beta_{T} l^{2} \Delta T/\left( {U_{0}^{2} x} \right)$ is Richardson number, $N_{r} = \frac{{\left( {\rho_{p} - \rho_{\infty } } \right)\Delta C}}{{\left( {1 - C_{\infty } } \right)\rho_{\infty } \beta \Delta T}}$ is Buoyancy ratio factor, $R_{b} = \frac{{\left( {\rho_{m\infty } - \rho_{\infty } } \right)\gamma_{1} \Delta n}}{{\left( {1 - C_{\infty } } \right)\rho_{\infty } \beta \Delta T}}$ is the Rayleigh number, $Pr = v/\alpha$ is Prandtl number, $N_{T} = \tau D_{T} \Delta T/\left( {vT_{\infty } } \right)$ is thermophoresis constraint, $N_{B} = \tau D_{B} \Delta C/\left( {vT_{\infty } } \right)$ is Brownian motion factor, $\zeta = Q_{0} l/\left( {\rho C_{p} U_{0} } \right)$ is heat generation/absorption term, $Lb = D_{B} /D_{n}$ is bioconvection Lewis number, $Sc = v/D_{B}$ is Schmidt number, $Pe$ is the Peclet number, $E = \frac{{E_{a} }}{{\kappa T_{\infty } }}$ is the activation energy and $\sigma = n_{\infty } /\left( {n_{w} - n_{\infty } } \right)$ is the motile parameter.

The physical interested quantities $\left( {C_{f} ,\,\,Nu_{x} ,\,\,Sh_{x} ,\,Nn_{x} } \right)$ are expressed as:

13 $$C_{f} = \frac{{2\tau_{w} }}{{\rho U_{w}^{2} }},\,\,\,Nu_{x} = \frac{{xq_{w} }}{k\Delta T},\,\,Nn_{x} = \frac{{xq_{n} }}{{D_{n} \Delta n}},\,\,\,Sh_{x} = \frac{{xq_{m} }}{{D_{B} \Delta C}}.\,$$

where $q_{w} = - k\left( {\partial T/\partial r} \right)_{r = a}$ is the heat flux at the surface, $\tau_{w} = - \mu \left( {\partial u/\partial r} \right)_{r = a} \,$ is the shear stress at the surface, $q_{n} = \, - D_{n} \left( {\partial n/\partial r} \right)_{r = a}$ is the motile microbes flux and $q_{m} = \, - D_{B} \left( {\partial C/\partial r} \right)_{r = a}$ is the mass flux.

The transformed from of Eq. (13) is:

14 $$\frac{1}{2}C_{f} \sqrt {Re_{x} } = - f^{\prime\prime}\left( 0 \right),\,\,\,\,\frac{{Sh_{x} }}{{\sqrt {Re_{x} } }} = - \varphi^{\prime}\left( 0 \right),\,\,\,\,\frac{{Nn_{x} }}{{\sqrt {Re_{x} } }} = - \hbar^{\prime}\left( 0 \right),\,\,\,\,\frac{{Nu_{x} }}{{\sqrt {Re_{x} } }} = - \theta^{\prime}\left( 0 \right).$$

where $\,Re_{x} = U_{0} x^{2} /vl$ is the local Reynolds number.

## Numerical solution

The detail explanation related to PCM methodologies are followed as^62–65^:

### Step 1

#### Generalization to 1st order ODE

15 $$\left. \begin{gathered} \lambda_{1} = f\left( \eta \right),\quad \lambda_{3} = f^{^{\prime\prime}} \left( \eta \right),\quad \lambda_{5} = \theta^{^{\prime}} \left( \eta \right),\quad \lambda = \lambda_{7} = \phi^{^{\prime}} \left( \eta \right),\quad \lambda_{9} = \hbar^{^{\prime}} \left( \eta \right) \hfill \\ \lambda_{2} = f^{^{\prime}} \left( \eta \right),\quad \lambda_{4} = \theta \left( \eta \right),\quad \lambda_{6} = \phi \left( \eta \right),\quad \lambda_{8} = \hbar \left( \eta \right). \hfill \\ \end{gathered} \right\}$$

By putting Eq. (15) in Eq. (8)–(11) & (12), we get:

16 $$\left( {2\gamma \eta + 1} \right)\lambda_{2}^{^{\prime}} + \left( {2\gamma + \lambda_{1} } \right)\lambda_{3} - \lambda_{2}^{^{\prime}} + Ri\left[ {\lambda_{4} - N_{r} \lambda_{6} - R_{b} \lambda_{8} } \right]\cos \alpha_{1} = 0$$

17 $$\left( {2\gamma \eta + 1} \right)\lambda_{5}^{^{\prime}} + \left( {2\gamma + Pr\lambda_{1} } \right)\lambda_{5} + \left( {2\gamma \eta + 1} \right)Pr\left[ {N_{B} \lambda_{7} + N_{T} \lambda_{7} } \right]\lambda_{7} + Pr\zeta \lambda_{4} = 0$$

18 $$\left( {2\gamma \eta + 1} \right)N_{B} \lambda_{7}^{^{\prime}} + N_{B} \left( {2\gamma + Sc\lambda_{1} } \right)\lambda_{7} + N_{T} \left( {\left( {2\gamma \eta + 1} \right)\lambda_{5}^{^{\prime}} + 2\gamma \lambda_{7} } \right) - Kr\left( {1 + \varepsilon \delta } \right)^{n} \lambda_{6} \;\exp \left( { - \frac{E}{1 + \varepsilon \delta }} \right) = 0$$

19 $$\left( {2\gamma \eta + 1} \right)\lambda_{9}^{^{\prime}} + 2\gamma \lambda_{9} + Lb\,Pr\lambda_{9} - Pe\left[ \begin{gathered} \sigma \gamma \lambda_{7} + \sigma \left( {2\gamma \eta + 1} \right)\lambda_{7}^{^{\prime}} + \gamma \lambda_{7} \lambda_{8} + \hfill \\ \left( {2\gamma \eta + 1} \right)\lambda_{7}^{^{\prime}} \lambda_{8} + \left( {2\gamma \eta + 1} \right)\lambda_{7} \lambda_{9} \hfill \\ \end{gathered} \right] = 0$$

With boundary Conditions

20 $$\begin{gathered} \lambda_{1} \left( 0 \right) = 0,\,\,\lambda_{2} \left( 0 \right) = 1,\,\,\lambda_{4} \left( 0 \right) = 1,\,\,\lambda_{6} \left( 0 \right) = 1,\,\,\lambda_{8} \left( 0 \right) = 1\;\;\eta \to \infty : \hfill \\ \lambda_{2} \to 0,\;\lambda_{4} \to 0,\;\lambda_{6} \to 0,\;\lambda_{8} \to 0 \hfill \\ \end{gathered}$$

### Step 2

#### Introducing parameter ***p*** in Eq. (16)–(20)

21 $$\left( {2\gamma \eta + 1} \right)\lambda_{3}^{^{\prime}} + \left( {2\gamma + \lambda_{1} } \right)\left( {\lambda_{3} - 1} \right)p - \lambda_{2}^{2} + Ri\left[ {\lambda_{4} - N_{r} \lambda_{6} - R_{b} \lambda_{8} } \right]\cos \alpha_{1} = 0$$

22 $$\left( {2\gamma \eta + 1} \right)\lambda_{5}^{^{\prime}} + \left( {2\gamma + Pr\lambda_{1} } \right)\left( {\lambda_{5} - 1} \right)p + \left( {2\gamma \eta + 1} \right)Pr\left[ {N_{B} \lambda_{7} + N_{T} \lambda_{7} } \right]\lambda_{7} + Pr\zeta \lambda_{4} = 0$$

23 $$\left( {2\gamma \eta + 1} \right)N_{B} \lambda_{7}^{^{\prime}} + N_{B} \left( {2\gamma + Sc\lambda_{1} } \right)\left( {\lambda_{7} - 1} \right)p + N_{T} \left( {\left( {2\gamma \eta + 1} \right)\lambda_{5}^{^{\prime}} + 2\gamma \lambda_{7} } \right) - Kr\left( {1 + \varepsilon \delta } \right)^{n} \lambda_{6} \,\,\exp \left( { - \frac{E}{1 + \varepsilon \delta }} \right) = 0$$

24 $$\left( {2\gamma \eta + 1} \right)\lambda_{9}^{^{\prime}} + 2\gamma \left( {\lambda_{9} - 1} \right)p + Lb\,Pr\lambda_{9} - Pe\left[ \begin{gathered} \sigma \gamma \lambda_{7} + \sigma \left( {2\gamma \eta + 1} \right)\lambda_{7}^{^{\prime}} + \gamma \lambda_{7} \lambda_{8} + \hfill \\ \left( {2\gamma \eta + 1} \right)\lambda_{7}^{^{\prime}} \lambda_{8} + \left( {2\gamma \eta + 1} \right)\lambda_{7} \lambda_{9} \hfill \\ \end{gathered} \right] = 0$$

## Results and discussion

This segment expresses the physical mechanisms and reason behind the increasing and decreasing effect of velocity, mass and energy outlines versus physical interest quantities. The following are some different profiles:

### Velocity interpretation

**Figure 2:**
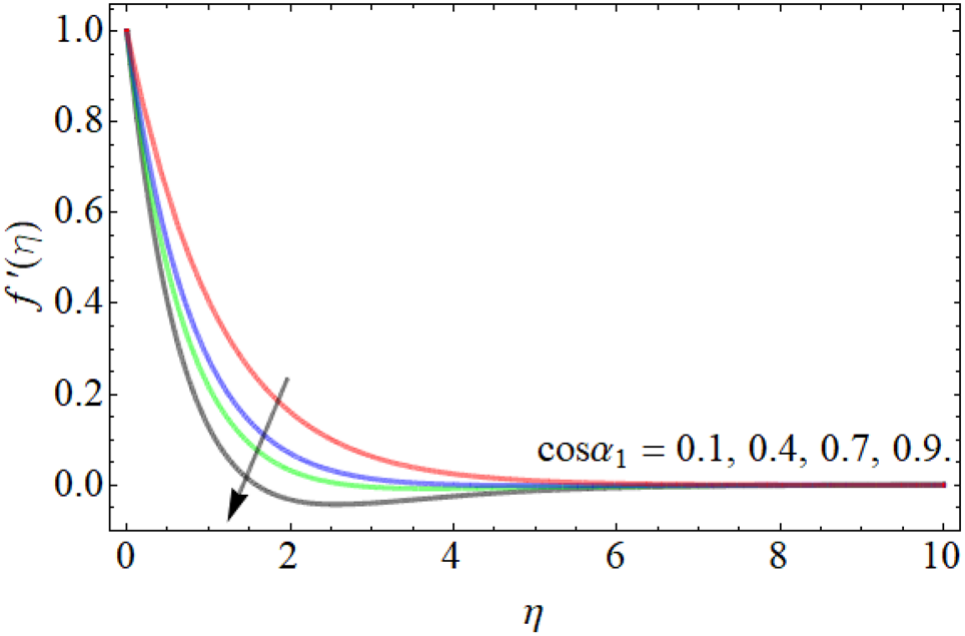
Velocity curve versus angle of inclination $\cos \alpha_{1}$.

**Figure 3:**
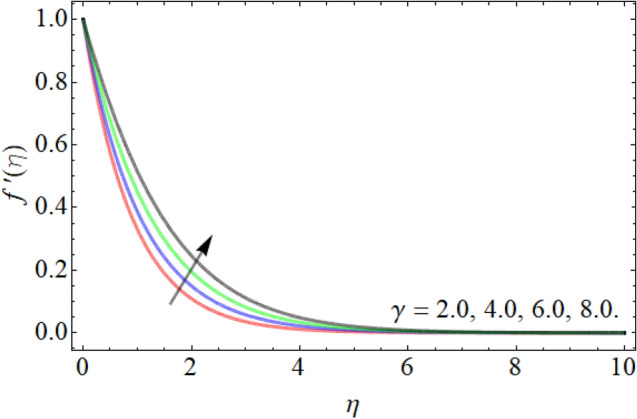
Velocity curve versus the curvature factor $\gamma$.

**Figure 4:**
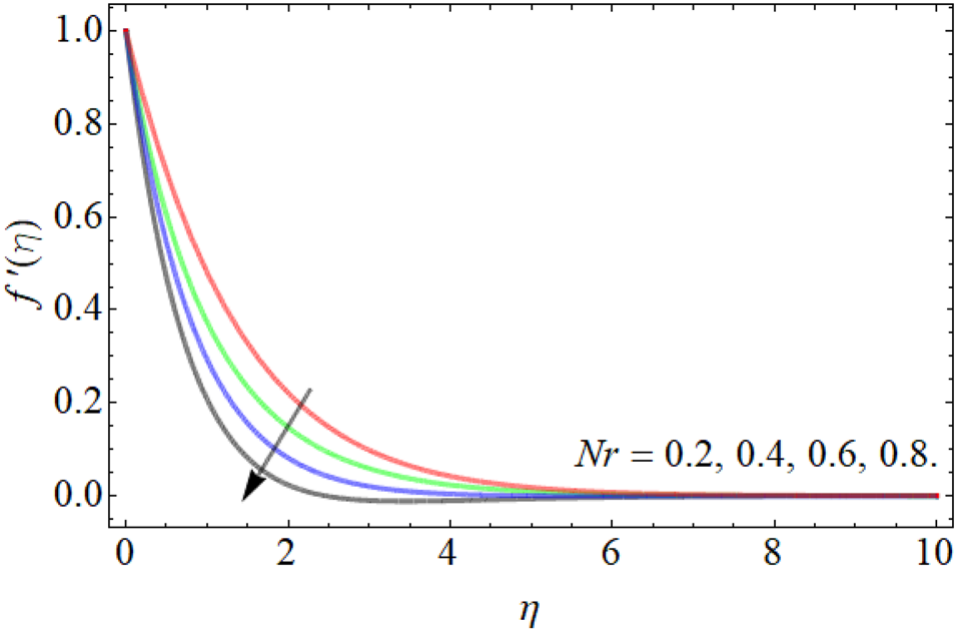
Velocity curve versus the buoyancy ratio term $Nr$.

**Figure 5:**
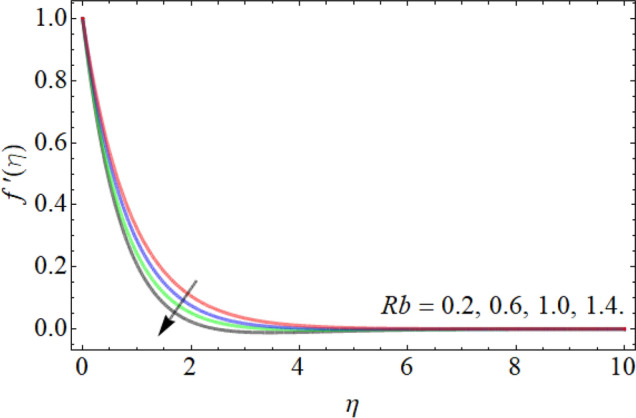
Velocity curve versus the bioconvection Rayleigh number $Rb$.

**Figure 6:**
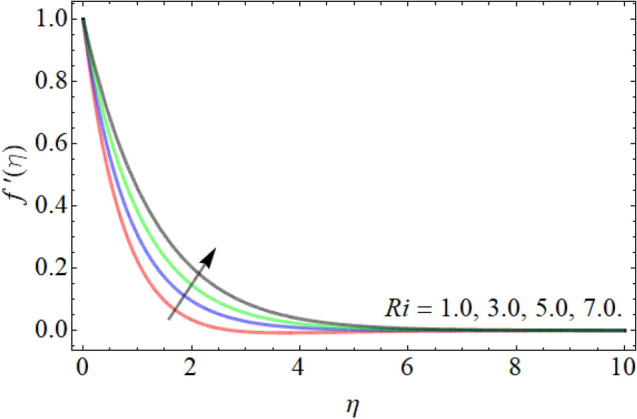
Velocity curve versus the Richardson number $Ri$.

Figures 2, 3, 4, 5, 6 explain the exhibition of velocity curves $f^{\prime}\left( \eta \right)$ versus the different values of inclination angle $\cos \alpha_{1}$, curvature factor $\gamma$, buoyancy ratio term $Nr$, $Rb$ and Richardson number $Ri$. Figure 2 revealed that the velocity decays with the influence of inclination angle $\cos \alpha_{1}$, while enhances against the variation of curvature factor $\gamma$. Physically, the flow velocity at plate surface is greater than the velocity at rough or inclined surface, that the rising angle inclination drops the fluid curve as publicized in Fig. 2. Figures 4 and 5 exhibit that the velocity curve also lessens with the flourishing effect of both buoyancy ratio term $Nr$ and bioconvection Rayleigh number $Rb$. Figure 6 exposed that the fluid velocity declines with the rising influence of Richardson number $Ri$. Physically, the factor $Ri$ is in direction proportion with the gravitational effect *g*. So, the rising effect of Richardson number augments the gravitational effect, which provides hurdle to the fluid flow.

### Energy interpretation

**Figure 7:**
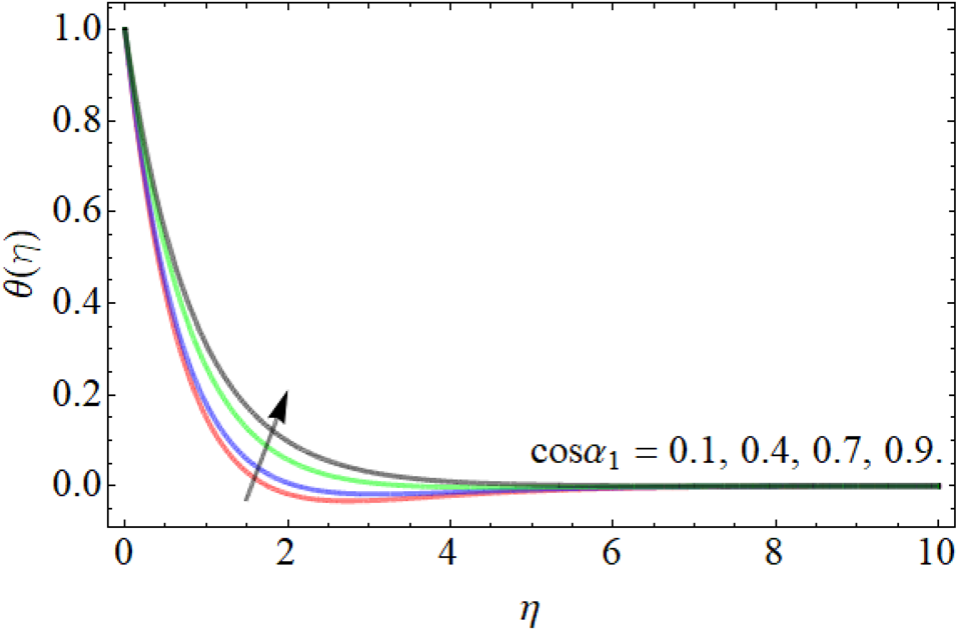
Energy outline versus the inclination angle $\cos \alpha_{1}$.

**Figure 8:**
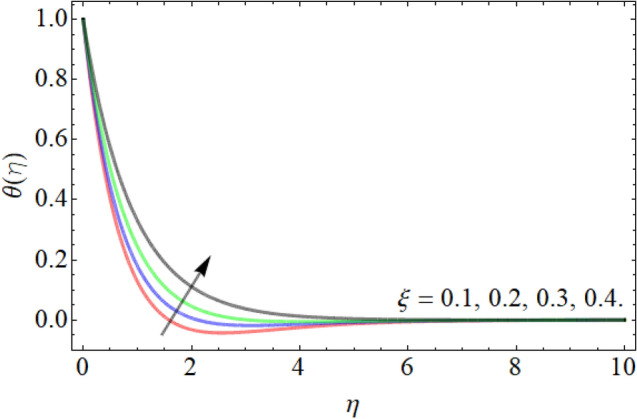
Energy outline versus the heat source term $\xi$.

**Figure 9:**
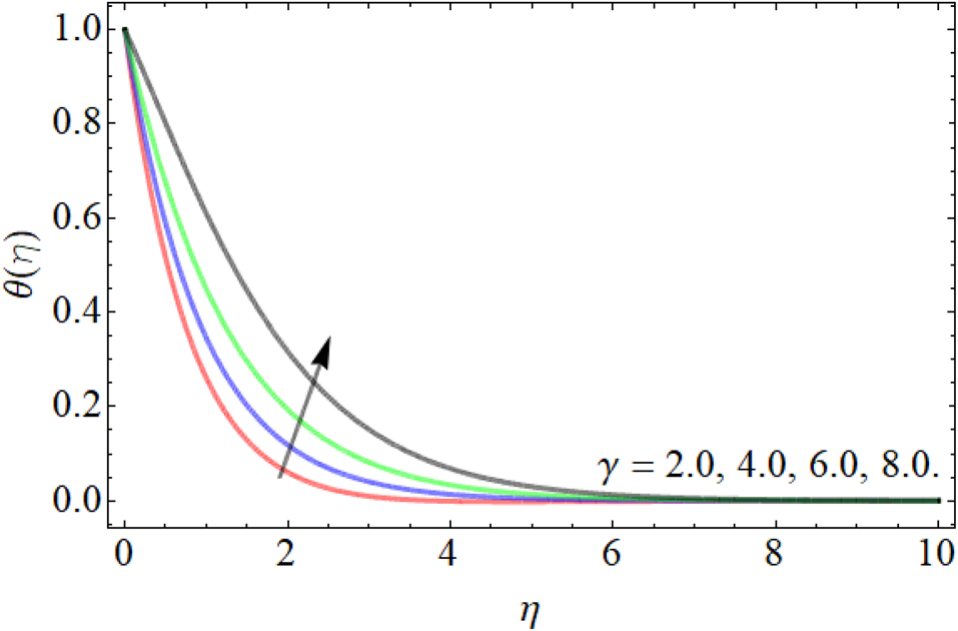
Energy outline versus the curvature factor $\gamma$.

**Figure 10:**
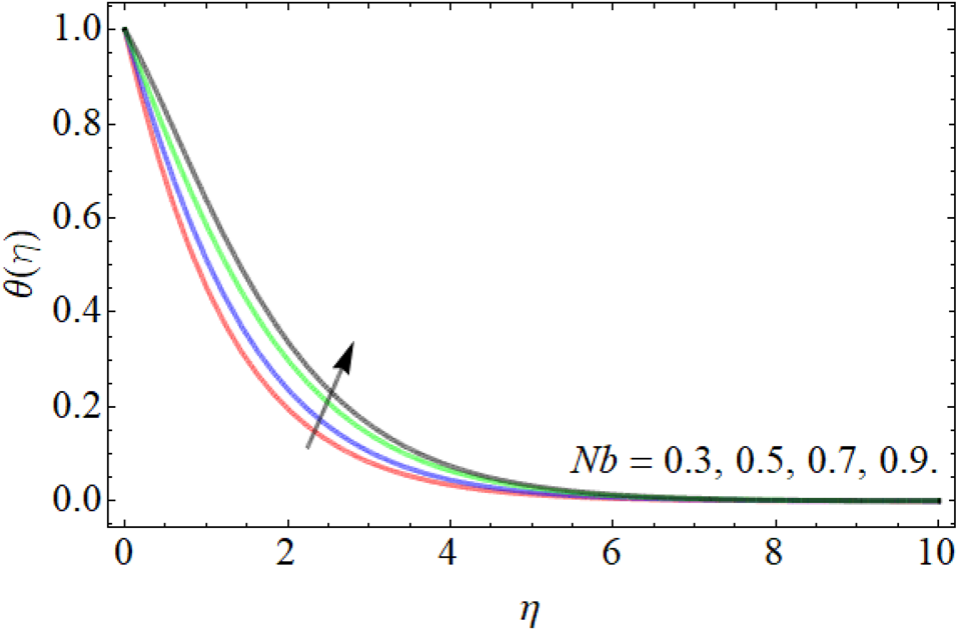
Energy outline versus the Brownian motion $Nb$.

**Figure 11:**
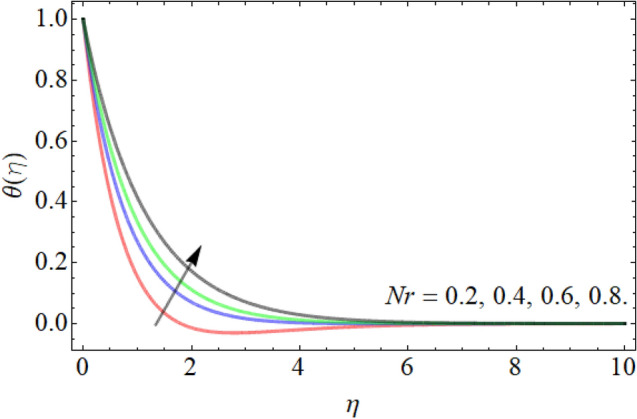
Energy outline versus the Buoyancy ration term $Nr$.

**Figure 12:**
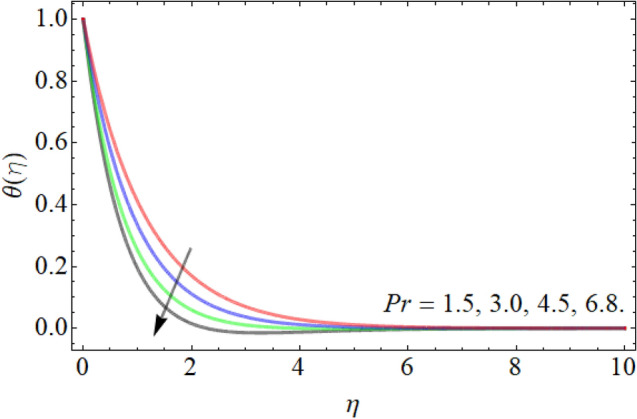
Energy outline versus the Prandtl number $Pr$.

**Figure 13:**
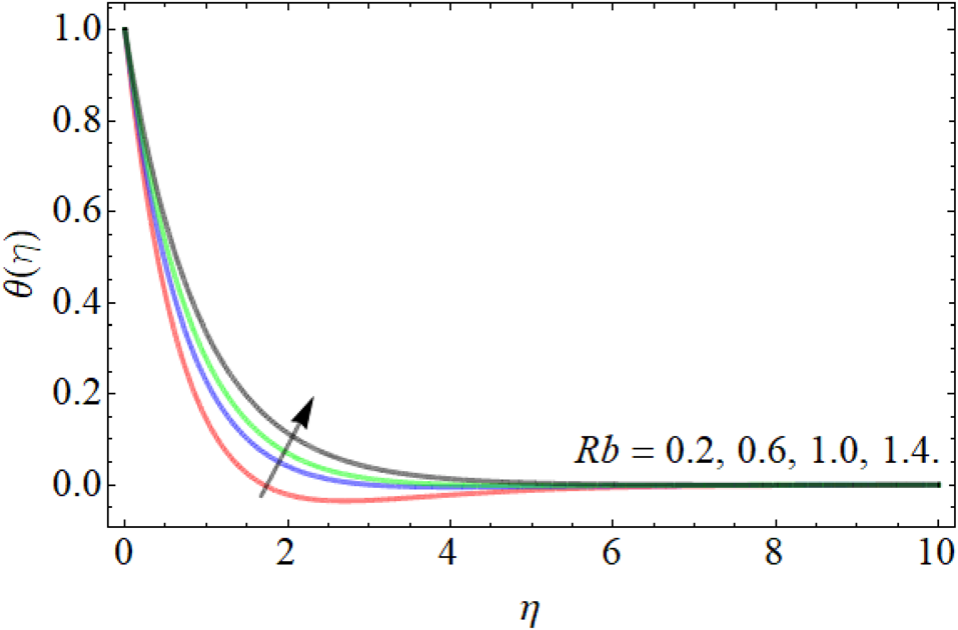
Energy outline versus the bioconvection Rayleigh number $Rb$.

**Figure 14:**
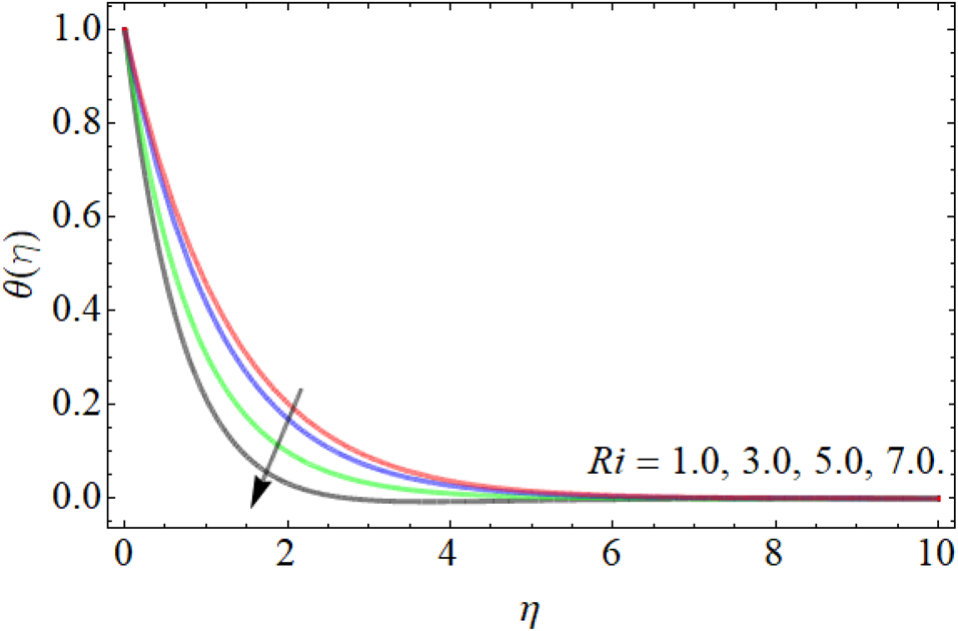
Energy outline versus the Richardson number $Ri$.

Figures 7, 8, 9, 10, 11, 12, 13, 14 describe the demonstration of energy outlines $\theta \left( \eta \right)$ versus the different values of inclination angle $\cos \alpha_{1}$, heat source term $\xi$, curvature factor $\gamma$, Brownian motion $Nb$, Buoyancy ration term $Nr$, Prandtl number $Pr$, Rayleigh number $Rb$ and Richardson number $Ri$ respectively. Figures 7 and 8 evaluated that the energy outline enhances with the consequence of inclination angle $\cos \alpha_{1}$ and heat source term $\xi$. As we have discoursed in the velocity profile that the rising inclination angle of stretching cylinder opposes to the fluid flow, that opposing force generates an extra heat, which causes the augmentation of heat profile as presented in Fig. 7. Similarly, the heat generation term diminishes the density and specific heat capacity of the fluid, which causes in the enhancement of energy outline as publicized in Fig. 8. Figures 9 and 10 reported that the influence of curvature term $\gamma$ and Brownian motion $Nb$ both accelerates the energy curve. Physically, the dissemination rate of fluid molecules augments with the effect of Nb, which results in the improvement of energy outline as presented in Fig. 10. Figures 11 and 12 illustrate that the energy curve develops with the upshot of $Nr$, while decays with the Prandtl number. Physically, higher Prandtl fluid has always higher kinetic viscosity and less thermal diffusivity and less Prandtl fluid has an opposite scene, that’s why, the effect of Prandtl number declines the energy curve as revealed in Fig. 12. Figures 13 and 14 demonstrate that the energy field inclines with the influence of $Rb$ and diminish versus the Richardson number $Ri$.

### Mass and microorganism interpretation

**Figure 15:**
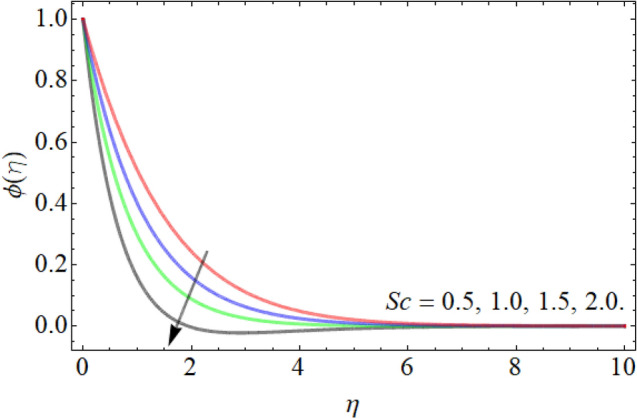
Exposition of mass outline versus the *Sc*.

**Figure 16:**
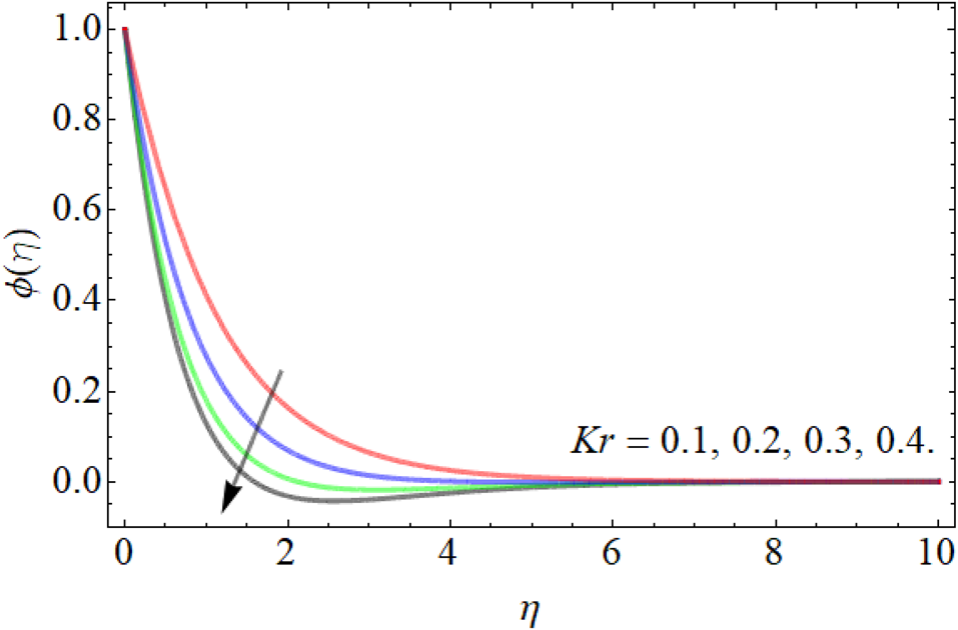
Exposition of mass outline versus the *Kr*.

**Figure 17:**
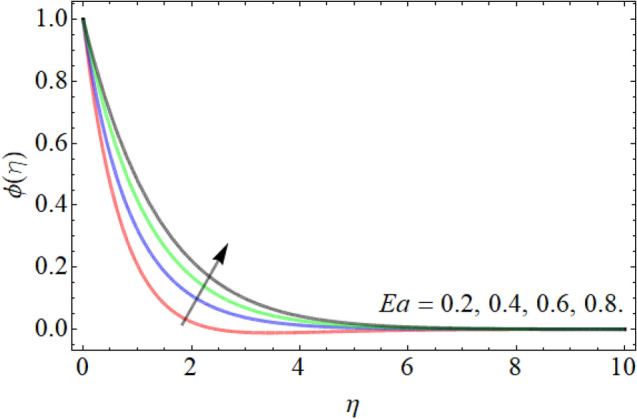
Exposition of mass outline versus the activation energy *Ea*.

Figures 15, 16, 17 designate the demonstration of mass outlines $\phi \left( \eta \right)$ versus *Sc*, *Kr* and *Ea* respectively. Figures 15 and 16 express that the mass field weakens with the outcome of *Sc* and *Kr.* The kinetic viscosity of fluid is directly proportion to the Schmidt number, while the diffusion rate of molecules has in inverse relation. That’s why, the influence of *Sc* drops the mass transmission rate as exposed in Fig. 15. Similarly, the influence of chemical reaction also lowers the fluid concentration outline, because the chemical reaction opposes to the particle movement, which results in the reduction of mass curve. Figure 17 highlighted that the effect of activation energy boosts the mass outline. It is known as the minimal energy required to energize or activate particles or atoms in order to participate in a chemical change or conversion. That’s why, the effect of activation energy boosts the mass transference rate as revealed in Fig. 17.

**Figure 18:**
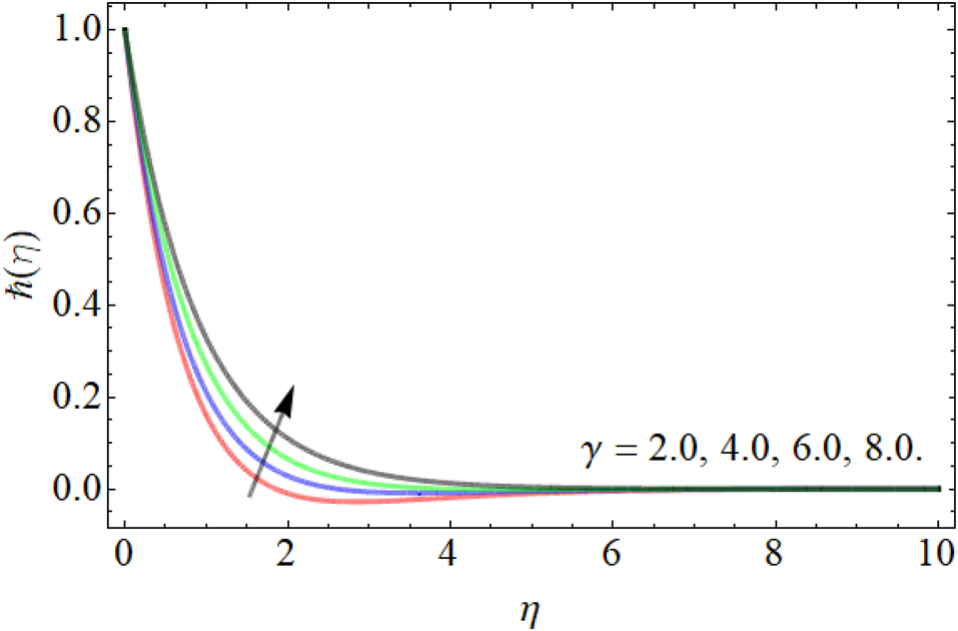
Exposition of motile microbes outline versus the curvature factor $\gamma$.

**Figure 19:**
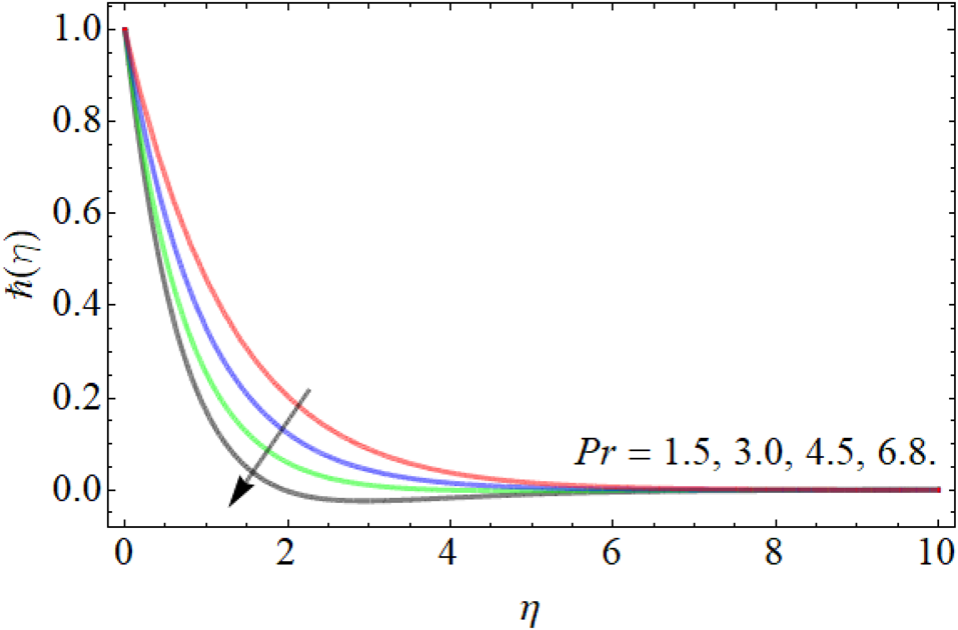
Exposition of motile microbes outline versus the Prandtl number *Pr*.

**Figure 20:**
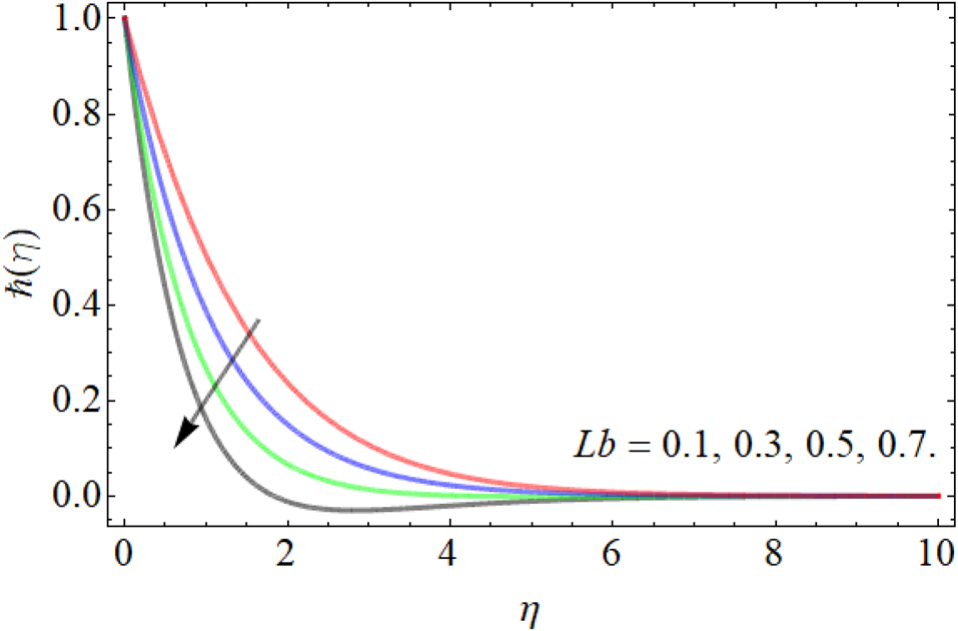
Exposition of motile microbes outline versus the bioconvection Lewis number *Lb*. Table 1 displays the numerical results for skin friction $- f^{\prime\prime}\left( 0 \right)$, Nusselt number $- \theta^{\prime}\left( 0 \right)$, Sherwood number $- \phi^{\prime}\left( 0 \right)$ and density of motile microorganism $- \hbar^{\prime}\left( 0 \right)$.

Figures 18, 19, 20 entitle the appearance of motile microorganism interpretation $\hbar \left( \eta \right)$ versus the different values of curvature factor $\gamma$, *Pr* and *Lb* respectively. Figures 18 and 19 show that the motile microbes curve enhances by the action of curvature factor $\gamma$, while declines with the upshot of Prandtl number *Pr.* Similarly, the influence of Lewis number also drops the motile microbe’s profile as revealed in Fig. 20.

**Table 1:** Numerical outcomes for physical interest quantities.

*Lb*	*Nb*	*Sc*	$\cos \alpha_{1}$	$- f^{\prime\prime}\left( 0 \right)$	$- \theta^{\prime}\left( 0 \right)$	$- \phi^{\prime}\left( 0 \right)$	$- \hbar^{\prime}\left( 0 \right)$
0.5	0.2	0.2	0.3	1.2330	0.1610	1.2155	1.4026
0.6				1.2330	0.1610	1.2155	1.5235
**0.7**				**1.2330**	**0.1610**	**1.2155**	**1.6245**
	0.2			1.2242	0.4495	0.0654	0.7515
	0.3			1.2232	0.4093	1.1062	0.7575
	**0.4**			**1.117**	**0.3358**	**1.1369**	**0.7657**
		0.2		1.2440	0.5970	0.3023	1.2920
		0.4		1.2422	0.5210	0.4131	1.3061
		**0.6**		**1.2332**	**0.4213**	**0.5759**	**1.3264**
			0.3	1.2461	0.1592	1.2126	1.3996
			0.707	1.2240	0.1748	1.2375	1.4264
			**0.860**	**1.2490**	**0.1697**	**1.1999**	**1.3965**

It can be observed that the Schmidt number enhances the Sherwood number. Similarly, the rising of inclination angle boosts the skin friction and Nusselt number.

Significant values are in bold.

## Conclusions

We have estimated numerically the nanoliquid boundary layer flow comprising gyrotactic microbes with mass and energy transmission across a stretching inclined cylinder. The consequences of chemical reaction, heat source, buoyancy force and Arrhenius activation energy is also considered on the nanofluid flow. The modeled equations are numerically calculated through the PCM approach. The significance findings are:

- The velocity curve drops with the influence of inclination angle $\cos \alpha_{1}$ and Richardson number $Ri$, while enhances against the variation of curvature factor $\gamma$.
- Rising effects of both buoyancy ratio term $Nr$ and $Rb$ diminish the velocity outline.
- The energy field boosts with the upshot of inclination angle $\cos \alpha_{1}$ and heat source term. while declines with the influence of Prandtl number and Richardson number $Ri$.
- Influence of curvature term $\gamma$, Buoyancy ratio term $Nr$ and $Nb$ accelerates the energy field.
- The mass profile diminishes with the effect of *Sc* and *Kr,* while the effect of activation energy boosts the mass outline.
- Motile microbes curve enhances by the action of curvature factor $\gamma$, while decays with the upshot of Prandtl number *Pr.* Similarly, the influence of Lewis number also drops the motile microbe’s profile.
- The proposed model may be further extended to the non-Newtonian case and can be handled through other numerical and analytical techniques.

## Data Availability

**“**Data will be available on demand from Bilal Ali”.
